# Impact of Preoperative Metal Patch Testing on Surgery Using Metal Implants

**DOI:** 10.1016/j.artd.2022.02.014

**Published:** 2022-03-21

**Authors:** Emi Sato, Akira Maeyama, Yutaro Yamasaki, Takuaki Yamamoto, Shinichi Imafuku

**Affiliations:** a Department of Dermatology, Fukuoka University Faculty of Medicine, Fukuoka, Japan; b Department of Orthopedic Surgery, Fukuoka University Hospital, Fukuoka, Japan

**Keywords:** Metal patch testing, Preoperative test, Metal allergic history, Metal implant surgery, Aseptic loosening

## Abstract

**Background:**

Patients scheduled for metal implant surgery in some facilities in Japan undergo preoperative metal patch testing (MPT). However, few studies have reported the impact of MPT results on scheduled surgery; therefore, the value of preoperative MPT remains unknown.

**Material and methods:**

In analysis 1,the preoperative MPT results requested by orthopedic surgeons from 4 institutions from 2014 to 2018 were retrospectively analyzed. In analysis 2, the medical records of all patients who underwent total hip arthroplasty, total knee arthroplasty, or total shoulder arthroplasty/reverse shoulder arthroplasty between 2014 and 2018 were collected. The number of patients who underwent MPT and their surgical results were analyzed.

**Results:**

In analysis 1, MPT was performed on 72 patients during the study period. The overall MPT positivity rate was 26.4% for the entire cohort in analysis 1. In 4 out of 19 MPT-positive cases, the results of MPT changed the treatment plan to use alternative materials or cancel the surgery. In analysis 2, 1087 patients underwent total hip arthroplasty, total knee arthroplasty, and TSA/RSA; only 16 patients underwent MPT. Aseptic loosening occurred postoperatively in 3 patients (0.3%), none of whom had a history of allergy, and none underwent preoperative MPT.

**Conclusion:**

Metal allergy did not appear to be directly involved in aseptic loosening to any large or meaningful degree in our patient cohort. Only 1.5% of the patients underwent preoperative MPT; therefore, our results suggest this testing had limited benefit or utility. Further studies are needed to determine whether MPT is necessary in preparation for joint replacement.

## Introduction

The so-called “metal allergy” usually represents a hypersensitive skin reaction due to metal components such as metal ornaments. Metal hypersensitivity is not rare globally; in particular, nickel hypersensitivity is relatively common, affecting 8%-18% of the general population in the European Union, even with the strict regulation of nickel content in ornaments and cosmetics that was introduced in 2005 [1]. Conversely, hypersensitivity reactions to surgical metal implants are extremely rare [2,3]. However, there are some reports of hypersensitivity to metal implants [[4], [5], [6]]. These reports raise concerns about the self-reported metal allergy history of patients scheduled to undergo metal implantation with nickel-containing materials in artificial joints. Therefore, surgeons often request preoperative metal patch testing (MPT) to exclude metal allergy. However, it remains unclear whether screening for a hypersensitivity reaction against metal ions on the skin adequately informs clinicians about hypersensitivity against metals implanted in bone. Inaccurate MPT results can result in an unnecessary change of treatment plan for patients with positive results. A literature search revealed that the significance of preoperative MPT in patients with self-reported metal allergies is unclear and lacks scientific evidence [7,8]. Furthermore, there is a small risk that the the MPT components may cause sensitization in rare cases [9,10]. The study aimed to investigate the value of preoperative MPT before orthopedic surgery using metal implants. We retrospectively reviewed the results of preoperative MPT performed in the Department of Dermatology, Fukuoka University Hospital (FUH), and tracked the impact of MPT results on subsequent treatment. We also reviewed data from all patients who underwent arthroplasty in our facility and investigated the significance of preoperative MPT based on a self-reported history of metal allergy.

## Material and methods

### Study design

#### Retrospective review of medical records

##### Patients

###### Analysis 1

Data from the preoperative MPT requested by the Department of Dermatology, FUH, from orthopedic surgeons at 4 institutions between 2014 and 2019 were extracted from the patient’s medical record database. The age, sex, self-reported metal allergy information, results of MPT, and surgical results of the patient were collected.

###### Analysis 2

To investigate the correlation between a history of metal allergy and postoperative orthopedic results, data from patients who underwent total hip arthroplasty (THA), total knee arthroplasty (TKA), and total shoulder arthroplasty/reverse total shoulder arthroplasty (TSA/RSA) at the Orthopedic Surgery Department, FUH, from 2014 to 2018 were extracted. The information collected included sex, age, MPT, MPT results, surgical procedure, and presence of aseptic loosening. Patients who underwent MPT in analysis 2 were also included in analysis 1.

##### Metal patch testing

“As is” metal is a small piece or powdered form of metal provided by the department that requested MPT. The 3 most common “as is” metals of orthopedic surgeons were zirconium alloy, cobalt-chromium alloys, and titanium alloy. “Metal series,” which contain 17 types of ionized metals, labeled by the Japanese authorities as an insured test, were purchased from Torii Pharmaceutical Co., Ltd. (Tokyo, Japan). The 17 metals in the metal series were aluminum chloride, cobalt chloride, tin dichloride, ferric chloride, hexachloroplatinic acid, palladium chloride, manganese chloride, indium trichloride, iridium tetrachloride, silver bromide, potassium dichromate, chromium sulfate, nickel sulfate, zinc chloride, tetrachloroauric acid, copper sulfate, and mercuric chloride. Patches were placed occlusively on the skin for 48 hours before being removed. Dermatologists confirmed the MPT results using the International Contact Dermatitis Result Grading standard at 48 hours, 72 hours, and 1 week after metal application. The patients were described as “positive” when the result of any metal in the “metal series” was positive.

##### Statistical analysis

Fisher’s exact test was used to compare data between the 2 groups, such as sex ratio, history of metal allergy, and whether a preoperative examination was performed. The chi-square test was used to compare the 3 types of MPT (“as is,” “metal series,” or “both”). The significance level was set at *P* < .05.

##### Institutional review board approval

The study protocol was approved by the Institutional Review Board of the Faculty of Medicine of Fukuoka University (approval no. U19-11-001). This study was conducted following the Declaration of Helsinki and the ethical guidelines for studies involving human subjects.

## Results

### Analysis 1

#### Demographic characteristics of the patients

A total of 72 patients (males, 14; females, 58) were referred to dermatologists for preoperative MPT by orthopedic surgeons in multiple institutions during the study period. The median age of the patients was 58 years, with no sex differences (male, 57 years; females, 58.5 years). The demographic characteristics of the patients are summarized in Table 1. Of the 72 patients, 67 (93.1%) had a self-reported history of metal allergies, and 5 (6.9%) had no history of metal allergy, but the orthopedic surgeon determined that they should undergo preoperative MPT due to skin symptoms such as eczema and urticaria.

**Table 1:** Patient demographic data.

Demographics	Total	Male	Female
N	72	14	58
Age at MPT			
Median	58	57	58.5
Q1, Q3	50.75, 70.75	52.25, 63.75	50.25, 73
Metal allergic history			
(+)	67	12	55
(−)	5	2	3

#### Positive rate of MPT

Table 2 summarizes the MPT results for the total cohort and the subgroups. The overall positive rate was 26.4% (19/72) for the total cohort, 14.3% (2/14) for males, and 29.3% (17/58) for females, with no significant sex differences (Fisher’s exact test, *P* = .33). Of the 67 patients with a history of metal allergy, 19 (28.4%) had a positive MPT, and of the 5 patients without a history of metal allergy, 0 (0%) had a positive MPT. However, because the population of patients without a history of metal allergy was too small, Fisher's exact test did not show significant findings (*P* = .32). When comparing the types of MPT, the positivity rates for “as is,” “metal series,” and “both” were found to be 5.4% (2/37), 33.3% (2/6), and 51.7% (15/29), respectively, with the rate of “as is” being significantly low. The “both” category includes patients who underwent both “as is” and “metal series” tests, and the result was described as positive when at least one of the tests was positive.

**Table 2:** Positive rate of metal patch testing in the total cohort and subgroups.

Subgroups	Total			Male			Female		
	N	(+), N	(+), %	N	(+), N	(+), %	N	(+), N	(+), %
	72	19	26.4	14	2	14.3	58	17	29.3
Metal allergic history									
(+)	67	19	28.4	12	2	16.7	55	17	30.9
(−)	5	0	0	2	0	0	3	0	0
Type of preoperative MPT									
As is	37	2	5.4^a^	7	0	0	30	2	6.7^a^
Metal series	6	2	33.3	2	0	0	4	2	50
Both	29	15	51.7	5	2	40	24	13	54.2

a *P* < .001 (chi-square).

#### Impact of MPT results on surgery

Table 3 shows the results of MPT and its impact on orthopedic surgery. Among the patients preoperatively tested, 19 had a positive result. In 4 of these cases, the result had an impact on the treatment policy, such as cancellation of surgery or change of materials. The other 15 patients were positive for metal sensitivity in the “metal series” unrelated to surgical materials. Specific metals with positive results but not associated with surgery include Ni, Co, Cr, Pd, Pt, Au, Ag, Zn, Sn, and Fe. All patients who tested positive for metals unrelated to these surgeries were operated on as scheduled, but none developed aseptic loosening or other adverse effects. The surgeries were changed in all 2 cases in which the patient tested positive for sensitivity to the “as is” metal. The planned surgery was changed due to a positive MPT result in 1 of 2 patients with a positive result in the “metal series” test and 1 of 15 patients (6.7%) with a positive result in the “both” test.

**Table 3:** Impact of metal patch testing on surgical decision-making.

Positive cases	Affected	Not affected	Total
As is	2	0	2
Metal series	1	1	2
Both	1	14	15
Total	4	15	19

#### Changes in surgical strategy due to MPT

Table 4 summarizes the details of the 4 patients whose MPT results affected surgical treatment. In one case, the scheduled operation (THA) was canceled. Surgical materials were changed in one patient for THA and in two patients for curved periacetabular osteotomy. The results of MPT in the 4 affected cases were reviewed based on clinical photographs; 2 cases were considered positive according to the International Contact Dermatitis Result Grading criteria based on photographs taken at 48 and 72 hours after MPT, while 2 were unclear. Of the 4 patients, 3 had a good postoperative course, and one had surgery canceled.

**Table 4:** Case series of patients with positive metal patch testing results.

Cases	Age, sex	Type of surgery	MPT type	Surgical status	Positive metal	Judgment based on ICDRG criteria using clinical photographs	Replaced surgical material	Postoperative status
Case 1	73, F	THA	As is	Performed	Cobalt-chromium alloy	+	Titanium	Good
Case 2	55, F	CPO	Both	Performed	Titanium alloy	IR	ETHIBOND EXCEL Ethicon, Inc. (Bridgewater, NJ)	Good
Case 3	34, F	CPO	Metal series	Performed	Cobalt chloride, potassium dichromate, nickel sulfate	Cobalt chloride (?) Potassium dichromate (?) Nickel sulfate (++)	Absorbent screw	Good
Case 4	60, F	THA	As is	Canceled	Zirconium alloy	IR	N/A	N/A

CPO, curved periacetabular osteotomy; IR, irritant reaction (false-positive); ICDRG, International Contact Dermatitis Result Grading; N/A, not applicable.

### Analysis 2

#### Relationship between metal allergy and aseptic loosening after arthroplasty

Table 5 summarizes the data of all 1087 patients (233 males and 854 females) who underwent THA, TKA, and TSA/RSA at our institution during the study period. The median age was 73 years (Q1, 65 years; Q3, 79 years), which was higher than the median age of 58 years in analysis 1. Only 16 patients (1.5%; 3 males and 13 females) with self-reported metal allergy underwent preoperative MPT. Of these 16 patients, 4 had a positive MPT result, but the surgical decision was modified in only 1 of the 4 cases (Table 4, case 1; case 4 was not included in the orthopedic surgical records because the THA was canceled). Of the 1087 patients who underwent THA, TKA, or TSA/RSA, only 3 (0.3%) had aseptic loosening after surgery; none of these 3 patients had a self-reported history of metal allergy or a positive preoperative MPT result. Infectious loosening occurred in 5 patients. Analysis of 5 cases of infectious loosening and 3 cases of aseptic loosening by unpaired t-test with Welch's correction showed that there was no significant difference in the time of onset between loosening due to infection and aseptic loosening, and the mean time was 71.20 days for infectious loosening and 215.7 days for aseptic loosening. Standard deviations were 76.61 for infectious loosening and 200.6 for aseptic loosening, respectively, with 95% confidence intervals of −23.93 to 166.3 and −282.6 to 713.9, respectively.

**Table 5:** Relationship between metal allergy and aseptic loosening after artificial joint replacement.

	THA, TKA, TSA/RSA from 2014 to 2018
Total, N	1087
Sex	
Male	233
Female	54
Age	
Median	73
Q1, Q3	65, 79
Metal allergic history	
(+)	16
(−)	1071

	Aseptic loosening		
With MPT	Total	Yes	No
Postive	4	0	4
Begative	12	0	12
w/o MPT	1071	3	1068

## Discussion

In this study, we reviewed 72 patients who were requested to undergo preoperative MPT by orthopedic surgeons from multiple institutions at the Department of Dermatology, FUH, and 1087 patients who underwent arthroplasty at the Department of Orthopedics, FUH, and analyzed the value of preoperative MPT based on a self-reported history of metal allergy.

We found only 28.4% of MPT-positive patients among those who reported metal allergy. The MPT positivity rate tended to be higher in the group of patients with self-reported metal allergy than in those with no history of metal allergy although the difference was not significant because there were only 5 patients with no history of metal allergy (Table 2). Frigerio et al. previously reported the results of a prospective study on MPT in 72 patients who underwent orthopedic metal implantation and showed that 5 patients who were preoperatively negative became positive 1 year after the surgery [11]. However, none of the 72 patients (including preoperatively positive and negative) had allergic symptoms, such as eczema or loosening after surgery [11]. Furthermore, 12 of 31 (39%) patients with a medical history of nickel allergy had negative MPT results, while 44 of 69 (5.8%) patients who did not report metal allergies had nickel hypersensitivity [11]. Although the frequency of nickel allergy was similar between the self-reported positive and negative groups, there was a nonnegligible discrepancy that can affect surgical decision-making [11]. In our study, the positive rate of preoperative MPT based on a self-reported history of metal allergy was 28.4% (19/67), which was significantly lower than that of MPT performed for other reasons, such as contact dermatitis, in the Department of Dermatology (64.8%, data not shown). Meanwhile, 54.5% of patients who did not declare metal allergy and underwent MPT in dermatology showed positive results (data not shown). These findings suggest that screening based on self-reported history of metal allergy may find more patients with metal allergies, but a nonnegligible proportion of patients may also have potential metal allergies. The benefits of MPT should be reconsidered for every patient with a history of metal allergy, without specific symptoms.

In a comparison of preoperative MPT by test type, “as is” testing had a low positive rate (5.4%), but positive “as is” test results had a large impact on surgical strategy in all 2 cases in our study. Because most of the metals that patients tested positive for were irrelevant to the contents of the implantation material, the results of the “metal series” or “both” tests were generally ignored. Although one patient tested positive for ionized cobalt and chromium in the “metal series” test, the surgery was performed as planned because the “as is” test showed negative results for cobalt-chromium alloy; this patient had metal implants for more than a year and had not experienced any adverse effects. The present findings suggest that surgeons who make the final decision regarding surgery place the greatest importance on the results of “as is” preoperative MPT. However, testing is not standardized and is sometimes difficult to judge.

Finally, we investigated whether a history of metal allergy symptoms on the skin affected the results of metal implant surgery. The pathogenic mechanism of aseptic loosening is that antigen-presenting cells that recognize the abrasion powder of the artificial joint as a foreign substance produce tumor necrosis factor-α, interleukin-1, and receptor activator of NF-kappaB ligand and induce osteoclasts to promote bone resorption and to create extra space around the artificial joint [12]. We started this research to determine whether confirmation of a delayed hypersensitivity reaction on the skin is an appropriate criterion to estimate the likelihood of an immune reaction around the artificial joint. Previous studies have reported that arthroplasty failure is unrelated to skin metal allergy [7,13]. Aseptic loosening of an artificial joint occurred in only 3 of 1087 patients (0.3%) treated in our orthopedic surgery department. None of these 3 patients had self-reported metal allergies or underwent preoperative MPT. The overall positive rate in this group (25%, 4/16) was much higher than that of aseptic loosening (0.3%), and it is less likely that aseptic loosening is induced by a reaction of cutaneous hypersensitivity to metal ions; however, this study has insufficient data to definitively answer this question. Larger clinical studies have reported similar findings. Cutaneous metal hypersensitivity was not associated with the surgical success rate in a case-control study of 356 patients definitively diagnosed with metal allergy by MPT among 70,698 patients undergoing THA [13] and a matched cohort study of 127 patients who underwent TKA after MPT [7]. In addition, some studies have reported that patients become sensitized to metal, especially after metal-on-metal implantation and implantation of a second implant with the same material [[14], [15], [16]], while Hallab et al. reported that metal allergies developed in <5% of sensitized patients and suggested that sensitization to metal implants does not necessarily lead to allergies [17]. Kwon et al. performed lymphocyte stimulation tests in patients with or without pseudotumor after metal-on-metal hip resurfacing and in control patients without any metal implants [18]; the nickel-positive rate was significantly higher in the group of patients with pseudotumor (80%) than that in the control group (13%) but did not differ between the group without a pseudotumor (45%) and the control group (13%). Furthermore, there have been several reports of cases in which MPT for metals used in implants became positive after surgery, but the symptoms of metal allergy spontaneously resolved with systemic administration of corticosteroids without metal implant removal [3,16]. Overall, the use of MPT as a screening or diagnostic test for hypersensitivity to metal implants is controversial, and a reliable method for the accurate diagnosis of allergies to metal implants has not been established [17,19]. Some studies favor lymphocyte stimulation testing over MPT for preoperative screening [18,20,21].

Despite these issues, from a clinical perspective, the surgical team prefers to request preoperative MPT because it helps to reduce the patient’s anxiety and avoid false accusations. However, inaccurate MPT results have a significant impact on treatment choices, which has resulted in the potential discouragement of necessary and safe surgery. Both surgeons and dermatologists should be aware that there is not yet enough scientific evidence that supports the claim preoperative screening by MPT in patients with metal allergies provides the benefit of avoiding loosening. More clinical data from more institutions on the success rate of metal implants with or without preoperative MPT may help us to conclude whether we should keep this controversial practice.

Our study has some limitations. First, this survey was conducted in a limited number of centers, and only a small number of patients underwent preoperative MPT. Second, it is unclear whether patients with positive MPT results have the same prognosis as those with negative results because none of the patients with positive preoperative MPT underwent scheduled surgery; however, aseptic loosening was exceptionally rare. Third, the accuracy of “as is” MPT is unknown.

## Conclusion

The main findings of our study are as follows. It is unclear whether preoperative MPT contributes to the success of metal implantation surgery. We are skeptical about the significance of preoperative MPT based on a patient-reported history of metal allergy. All physicians should carefully evaluate the MPT results because these results play an important role in surgical decision-making. Clinicians should remember that false positives lead to the avoidance of necessary surgery and are linked to a poor quality of life for affected patients.

## Acknowledgments

The authors thank Kelly Zammit, BVSc, from Edanz Group (https://en-author-services.edanz.com/ac) for editing a draft of this manuscript. They would also like to thank Editage (www.editage.com) for English language editing.

## Conflicts of interest

The authors declare that there are no conflicts of interest.

## Figures and Tables

**Conflict of Interest Statement for Maeyama:**
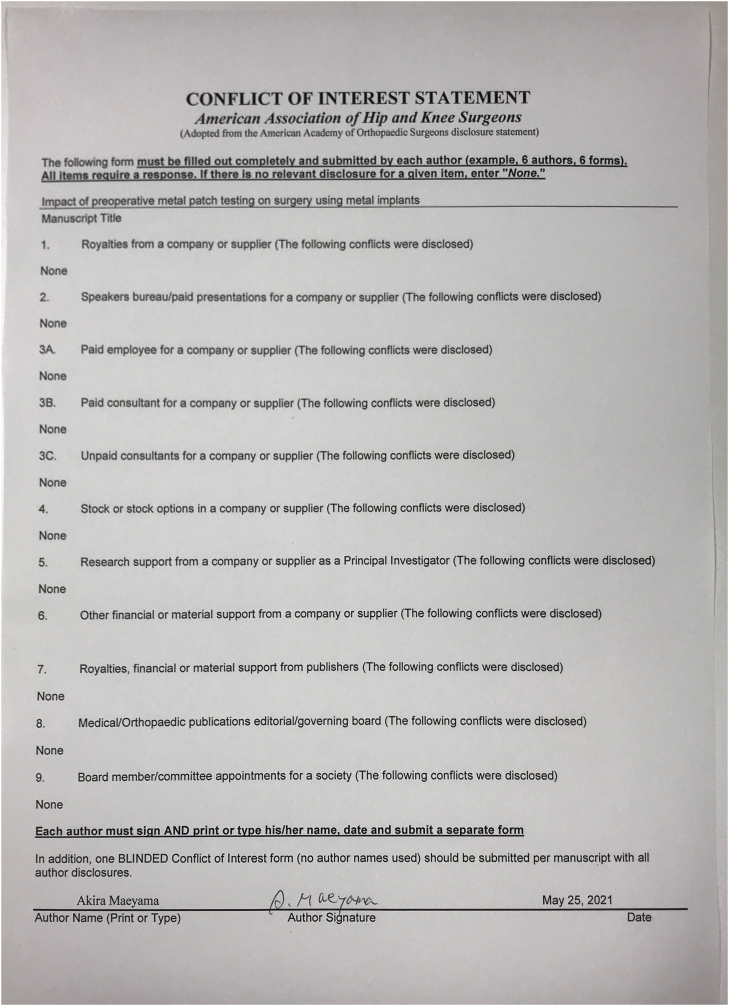


**Conflict of Interest Statement for Yamasaki:**
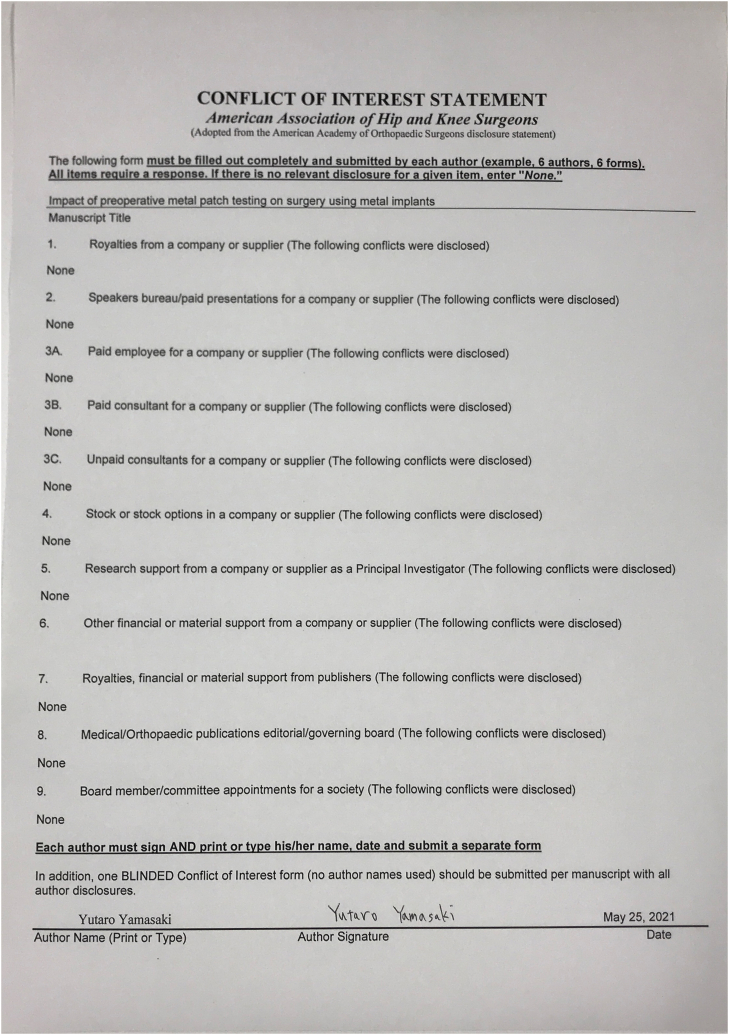


**Conflict of Interest Statement for Yamamoto:**
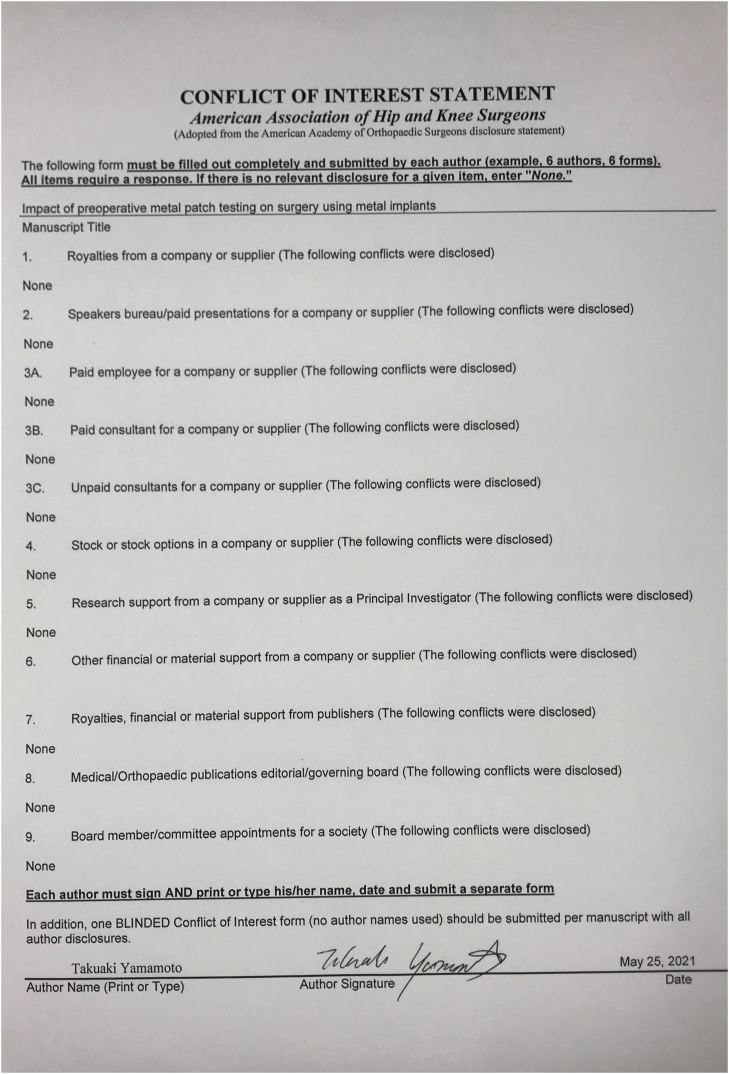


**Conflict of Interest Statement for Imafuku:**
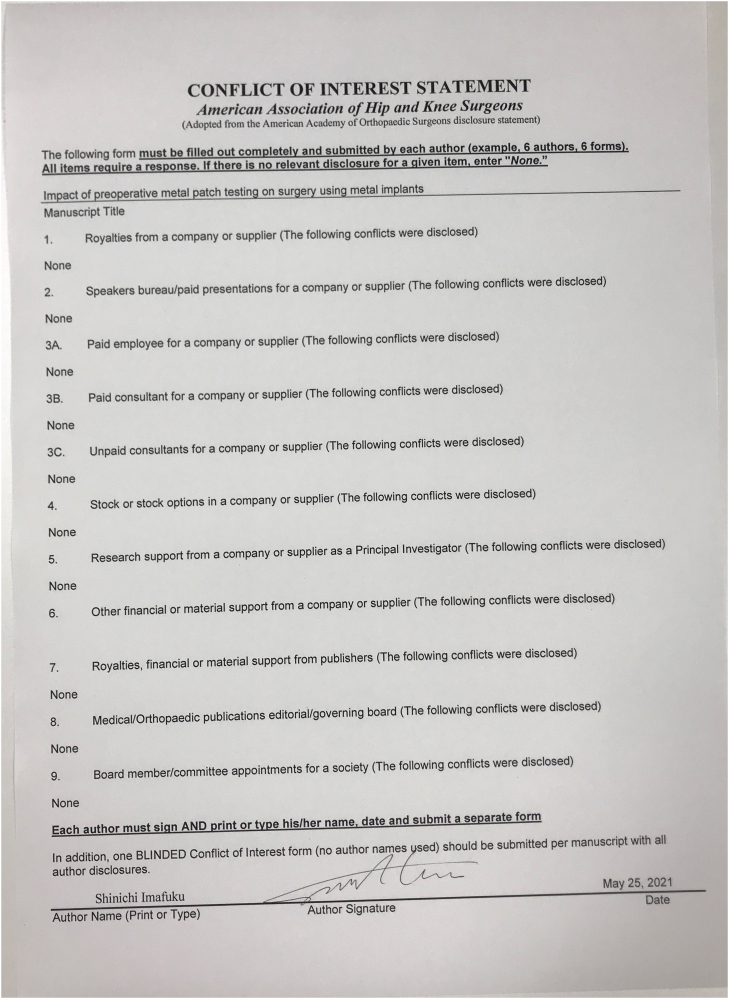


## Appendix A Supplementary data

Conflict of Interest Statement for Sato
